# Left Atrial Thrombus in Patients With Non-valvular Atrial Fibrillation: A Cross-Sectional Study in China

**DOI:** 10.3389/fcvm.2022.827101

**Published:** 2022-05-02

**Authors:** Shaobo Shi, Qingyan Zhao, Tao Liu, Shujuan Zhang, Jinjun Liang, Yanhong Tang, Bo Yang, He Huang, Congxin Huang

**Affiliations:** ^1^Department of Cardiology, Renmin Hospital of Wuhan University, Wuhan, China; ^2^Cardiovascular Research Institute, Wuhan University, Wuhan, China; ^3^Hubei Key Laboratory of Cardiology, Wuhan University, Wuhan, China

**Keywords:** atrial fibrillation, left atrial thrombus, prevalence, factors, transesophageal echocardiography

## Abstract

**Background:**

Stroke is predominately attributed to left atrial thrombus (LAT) in patients with non-valvular atrial fibrillation (NVAF), however, its detection rate in real clinical practice has been few reported in China.

**Objective:**

This study aimed to investigate the prevalence and associated factors of LAT in patients with NVAF in China.

**Methods:**

All adult NVAF patients undergoing transesophageal echocardiography (TEE) in the China Atrial Fibrillation Center database from January 2017 to January 2022 were enrolled in this study. The prevalence of LAT was calculated, and associated factors were identified.

**Results:**

A total of 36,007 NVAF inpatients from 602 hospitals in 30 provinces/autonomous regions/municipalities were included in the final analysis, with a median age of 66 years and 39.4% were female. LAT was present in 1,467 (4.1%) patients overall, 2.7, 5.7, and 6.8% in patients with paroxysmal, persistent, and long-standing persistent AF, respectively. In subgroup analysis, including age ≥ 65 years, CHA_2_DS_2_-VAS_C_ score ≥ 2, left atrial diameter (LAD) ≥ 50 mm, left ventricular ejection fraction (LVEF) < 50%, and anticoagulation, patients with paroxysmal AF always had the lowest LAT prevalence, followed by patients with persistent and long-standing persistent AF. Patients treated with anticoagulants had less prevalent LAT than those without anticoagulation (2.1 vs. 5.0%, p < 0.001). In multivariate analysis, AF pattern (both persistent AF and long-standing persistent AF), hypertension, chronic heart failure, coronary heart disease, transient ischemic attack/stroke, diabetes mellitus, and LAD (per 5 mm) were associated with an increased prevalence of LAT. However, LVEF (per 5%) and anticoagulation were associated with a reduced prevalence of LAT.

**Conclusion:**

LAT was found in 4.1% of Chinese adult NVAF inpatients underwent TEE in real-world experience. The prevalence of LAT mainly associated with non-paroxysmal AF, cardiovascular diseases, diabetes mellitus, enlarged left atrium, lower LVEF, and lack of anticoagulation therapy.

## Introduction

Atrial fibrillation (AF) is the most common sustained tachyarrhythmia. Our latest national cross-sectional epidemiological study found the prevalence of AF was 1.6% in the Chinese adult population, which indicates a 146% increase compares with the previous investigation in 2004 (1). AF has been proved to increased the risk of stroke and extracranial systemic embolism events, and thromboembolic events are associated with increased long-term disability and death (2). In turn, thrombosis and stroke prophylaxis are the cornerstones of AF therapy (3). AF-related embolism is predominately attributed to left atrial thrombus (LAT), which can be detected with transesophageal echocardiography (TEE), computed tomography, and magnetic resonance imaging (4). The management strategy for AF should be changed once LAT has been detected, such as strengthening anticoagulant therapy and delaying operation. Several reports have described the prevalence and characteristics of LAT in North America and Europe, and approximately 5–27% of AF patients without anticoagulation therapy (5). Even in anticoagulated patients with AF, LAT cannot be ignored due to the high incidence (6, 7), with a pooled prevalence of 2.73% in whom continuously anticoagulated for at least 3 weeks (8). However, few reports are available about the LAT prevalence in AF patients based on multicenter and large-sample research in China. The aim of this study was to determine the prevalence of LAT and explore its associated factors in inpatients with non-valvular AF (NVAF) from hospitals constructed the China Atrial Fibrillation Center.

## Methods

### Study Population

We conducted a real-world study of Chinese atrial fibrillation (RWS-CAF), which was a multicenter, observational, retrospective cohort study (9). The RWS-CAF collected medical records of patients with AF regularly from hospitals registered at the China Atrial Fibrillation Center (https://www.china-afc.org/). The center is a national regionalized non-profit academic organization that aims to promote, conduct and monitor standardized management of patients with AF in China.

In this study, we conducted a cross-sectional investigation on the prevalence of LAT in inpatients with NVAF, who aged 18 years or older and had undergone TEE examination from January 2017 to January 2022. Patients who lacked detailed medical documentation or had duplicated records were excluded. Only paroxysmal AF, persistent AF, and long-standing persistent AF were included in this study, and permanent AF was excluded due to the lack of exact AF duration. The definition of paroxysmal, persistent, and long-standing persistent AF was according to 2020 ESC Guidelines for the diagnosis and management of AF (3). We adhered to the STROBE statement for reporting observational studies. The registry is being conducted in accordance with the principles of the Declaration of Helsinki, local regulatory requirements, and clinical practice guidelines (registration number: ChiCTR1900021250). This study was approved by the Institutional Committee on Human Research at Renmin Hospital of Wuhan University (Wuhan, China). Written informed consent was waived due to retrospective analysis of electronic medical records and the patient private information was de-identified.

### Collection of Data

All patient demographics, medical history, laboratory data, echocardiographic data (included transthoracic echocardiography and TEE), and treatments were obtained retrospectively from medical records in the China Atrial Fibrillation Center. Information on anticoagulant drugs were collected on admission, so as to unify the criteria for evaluating their relationship with LAT. Laboratory data were collected and obtained from the most recent results prior to the TEE. The primary endpoint was the presence of LAT on TEE. The LAT includes LA/left atrial appendage (LAA) thrombus and LAA sludge. LA/LAA thrombus was defined as a fixed or mobile, well-circumscribed echogenic mass with a unique echotexture contrasting with the adjacent or underlying myocardium. LAA sludge was defined as a stationary echodensity within the LAA that did not fulfill the criteria for a thrombus (10). CHA_2_DS_2_-VASc Score was calculated from the sum of the risk factors of congestive heart failure, hypertension, age ≥ 75 years, diabetes mellitus, stroke, vascular disease, age 65–74 years, and sex category (female); weighing each by 1 except for stroke and age ≥ 75 years, which were weighed by 2 (11). Diagnosis of diseases, such as hypertension, chronic heart failure, coronary heart disease, transient ischemic attack (TIA), stroke, diabetes mellitus, etc., was determined based on recent management guidelines or expert consensus.

### Patient and Public Involvement

Patients and public were not involved in the design, conduct, reporting or dissemination of this research.

### Statistical Analysis

Data analysis was performed using SPSS V.19.0 software (IBM, West Grove, Pennsylvania, USA), and statistical significance was defined as a *P* < 0.05 with two-sided testing. Continuous variables are presented as the mean ± standard deviation (SD) or median (interquartile range [IQR]), and categorical variables are presented as counts and percentages (%). Means or medians were compared with Student's *t*-test, Wilcoxon rank-sum tests, and frequencies with chi-square analysis or Fisher's exact test for small numbers, as appropriate. Multivariate logistic regression with stepwise model was performed to estimate the associated factors for LAT. The results from this process were applied using a *P*-value of 0.01 to enter and 0.05 to stay in the model. The cases missing biomarker data were excluded by listwise with statistics software. Statistical charts were generated using Prism 7 (GraphPad) and Excel 2010 software.

## Results

### Patient Characteristics

**Figure 1:**
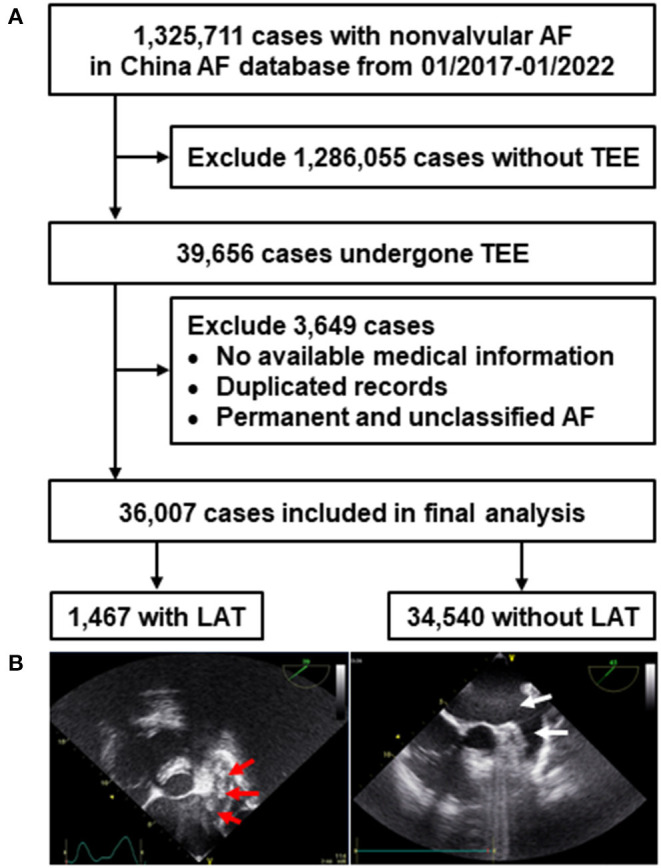
Flowchart of patients selection **(A)** and representative TEE images **(B)**. Red arrow in the left image indicated multiple LAT in a case with NVAF, white arrow in the right image showed no LAT. NVAF, non-valvular atrial fibrillation; LAT, left atrial thrombus; TEE, transesophageal echocardiography.

A total of 1,325,711 hospitalized patients with NVAF were screened initially from the China Atrial Fibrillation Center database between January 2017 and January 2022. Figure 1 depicts the flowchart for patient selection and representative TEE images. Briefly, after excluding patients who did not undergo TEE (*n* = 1,286,055), patients without available medical information and duplicated records (*n* = 1,014), and permanent and unclassified AF (*n* = 2,635). Overall, 36,007 NVAF patients from 602 hospitals in 30 provinces/autonomous regions/municipalities were included in the final analysis.

The clinical characteristics of the included patients on admission and before undergoing TEE are summarized in Table 1. In these included patients, the median age was 66 years, 39.4% were female, 20,135 (55.9%) patients had paroxysmal AF, 14,187 (39.4%) had persistent AF, and 1,685 (4.7%) had long-standing persistent AF. The mean CHA_2_DS_2_-VASc score was 2.6, and 72.0% of patients had a CHA_2_DS_2_-VASc score ≥ 2. The most common comorbidity was hypertension (55.9%), followed by chronic heart failure as New York Heart Association class II or higher (29.4%), coronary heart disease (18.6%), TIA/stroke (16.4%), diabetes mellitus (16.3%), vascular disease (10.5%), chronic obstructive pulmonary disease (COPD) (4.0%), cardiomyopathy (2.9%), hypothyroidism (1.9%), hyperthyroidism (1.7%), peripheral embolism (1.0%), and obstructive sleep apnea (0.7%). There were 30.9% (11,144) of included patients administrated with anticoagulants on admission. The median left atrial diameter (LAD) was 40 mm, and the median left ventricular ejection fraction (LVEF) was 60%.

**Table 1:** Baseline characteristics.

**Variable**	**All Patients**	**LAT**	**No LAT**	***P*** **-value**
	**(*n* = 36,007)**	**(*n* = 1,467)**	**(*n* = 34,540)**	
Age, years	66 (58–72)	66 (58–71)	66 (58–72)	0.882
Gender, female	14,183 (39.4)	566 (38.6)	13617 (39.4)	0.530
**AF pattern**
Paroxysmal	20,135 (55.9)	545 (37.2)	19,590 (56.7)	<0.001
Persistent	14,187 (39.4)	808 (55.1)	13,379 (38.7)	
Long-standing persistent	1,685 (4.7)	114 (7.8)	1,571 (4.5)	
**CHA**_**2**_ **DS**_**2**_ **-VASc Score**	2.6 ± 1.7	2.9 ± 1.6	2.6 ± 1.7	<0.001
0	3,398 (9.4)	82 (5.6)	3,316 (9.6)	<0.001
1	6,683 (18.6)	212 (14.5)	6,471 (18.7)	
≥2	25,926 (72.0)	1,173 (80.0)	24,753 (71.7)	
**Medical history**
Hypertension	20,127 (55.9)	890 (60.7)	19,237 (55.7)	<0.001
Chronic heart failure	10,599 (29.4)	577 (39.3)	10,022 (29.0)	<0.001
Coronary heart disease	6,707 (18.6)	307 (20.9)	6,400 (18.5)	0.021
TIA/Stroke	5,914 (16.4)	308 (21.0)	5,606 (16.2)	<0.001
Diabetes mellitus	5,862 (16.3)	273 (18.6)	5,589 (16.2)	0.015
Vascular disease	3,765 (10.5)	201 (13.7)	3,564 (10.3)	<0.001
COPD	1,427 (4.0)	64 (4.4)	1,363 (3.9)	0.415
Cardiomyopathy	1,056 (2.9)	70 (4.8)	986 (2.9)	<0.001
Hypothyroidism	688 (1.9)	45 (3.1)	643 (1.9)	0.002
Hyperthyroidism	618 (1.7)	22 (1.5)	596 (1.7)	0.600
Peripheral embolism	366 (1.0)	78 (5.3)	288 (0.8)	<0.001
Obstructive sleep apnea	254 (0.7)	8 (0.5)	246 (0.7)	0.631
**On admission**
Anticoagulants	11,144 (30.9)	233 (15.9)	10,911 (31.6)	<0.001
DOACs	6,820 (18.9)	68 (4.6)	6,752 (19.5)	<0.001
VKA	4,324 (12.0)	165 (11.3)	4,159 (12.1)	0.388
**Before TEE**
SBP, mmHg	129 (117–141)	130 (118–140)	129 (117–142)	0.698
DBP, mmHg	80 (70–88)	80 (71–90)	80 (70–88)	<0.001
LAD, mm	40 (36–45)	43 (38–49)	40 (36–45)	<0.001
LVEF, %	60 (54–65)	58 (50–64)	60 (55–65)	<0.001
INR	1.08 (0.99–1.23)	1.12 (1.01–1.41)	1.08 (0.99–1.23)	<0.001
Creatinine, μmol/L	77 (64–92)	78 (65–95)	77 (64–92)	0.008
CrCl, mL/min	74 (55–94)	73 (55–92)	74 (55–94)	0.511
NT-proBNP, μg/mL	774 (256–2013)	1,230 (584–3,131)	750 (249–1,971)	<0.001

*Values are median (interquartile range), n (%), or mean ± standard deviation. AF, atrial fibrillation; CHA_2_DS_2_-VASc, Congestive heart failure, Hypertension, Age ≥ 75 years, Diabetes mellitus, Stroke, Vascular disease, Age 65–74 years, Sex category (female); COPD, chronic obstructive pulmonary disease; CrCl, creatinine clearance; DBP, diastolic blood pressure; DOACs, direct oral anticoagulants; INR, international normalized ratio; LAD, left atrial diameter; LAT, left atrial thrombus; LVEF, left ventricular ejection fraction; NT-proBNP, N-terminal pro brain natriuretic peptide; SBP, systolic blood pressure; TEE, transesophageal echocardiography; TIA, transient ischemic attack; VKA, vitamin K antagonist*.

### Characteristics of Patients With LAT

Overall, LAT was detected in 1,467 (4.1%) inpatients with NVAF who underwent TEE. A comparison of the clinical characteristics between patients with and without LAT is presented in Table 1. Patients with LAT had a higher proportion of persistent AF and long-standing persistent AF, higher CHA_2_DS_2_-VASc score, more often a history of hypertension, chronic heart failure, coronary heart disease, TIA/stroke, diabetes mellitus, vascular disease, cardiomyopathy, hypothyroidism, and peripheral embolism (all *P* <0.05). The proportion of anticoagulants and direct oral anticoagulants use was lower in patients with LAT than in those without LAT (all *P* < 0.001) on admission. Notably, patients with LAT had a larger LAD, lower LVEF, and elevated international normalized ratio (INR), creatinine, and N-terminal pro brain natriuretic peptide (NT-proBNP) levels (all *P* < 0.01).

### Prevalence of LAT

**Figure 2:**
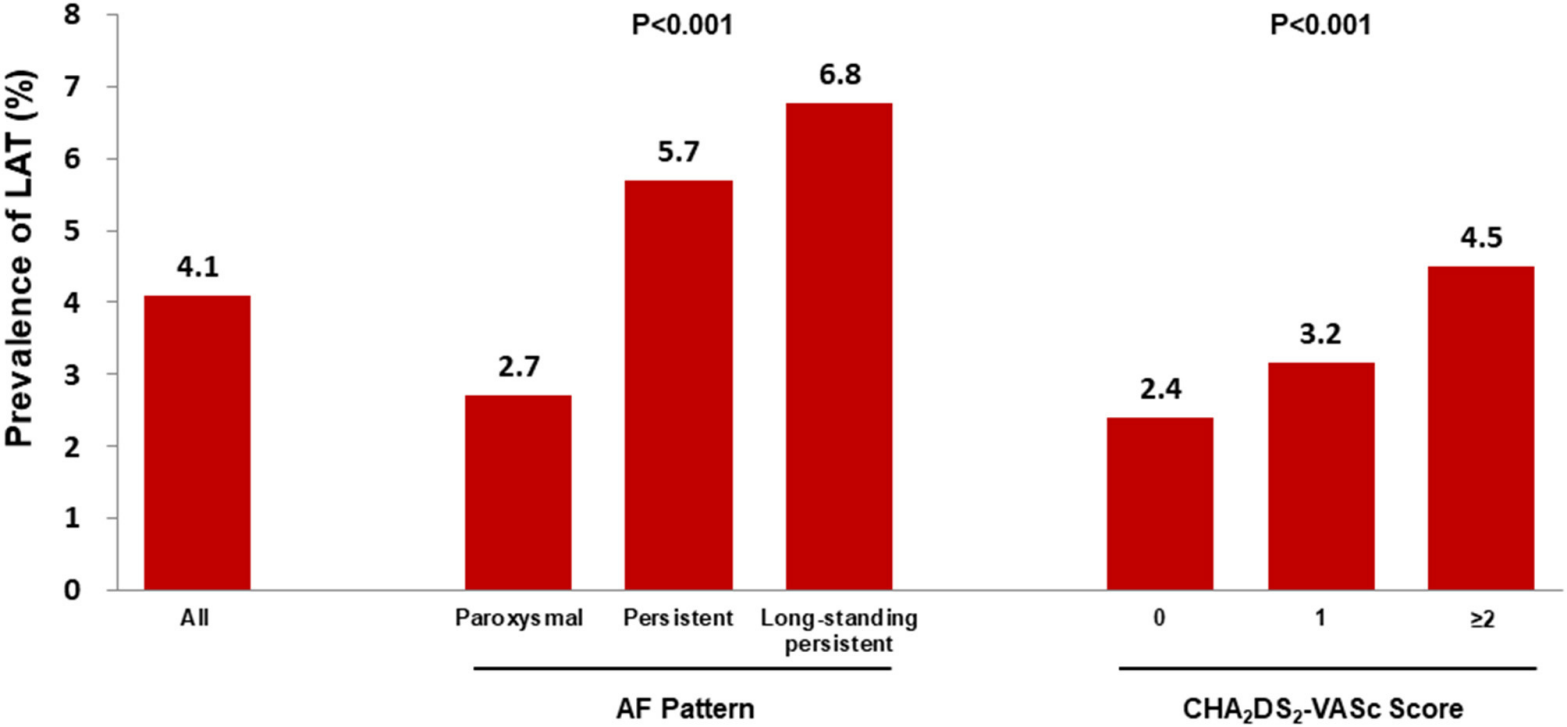
The prevalence of left atrial thrombus according to AF pattern and CHA_2_DS_2_-VASc score. AF, atrial fibrillation; CHA_2_DS_2_-VASc, Congestive heart failure, Hypertension, Age ≥ 75 years, Diabetes mellitus, Stroke, Vascular disease, Age 65–74 years, Sex category (female); LAT, left atrial thrombus. *P* < 0.001 indicated a significant statistical difference among groups.

**Figure 3:**
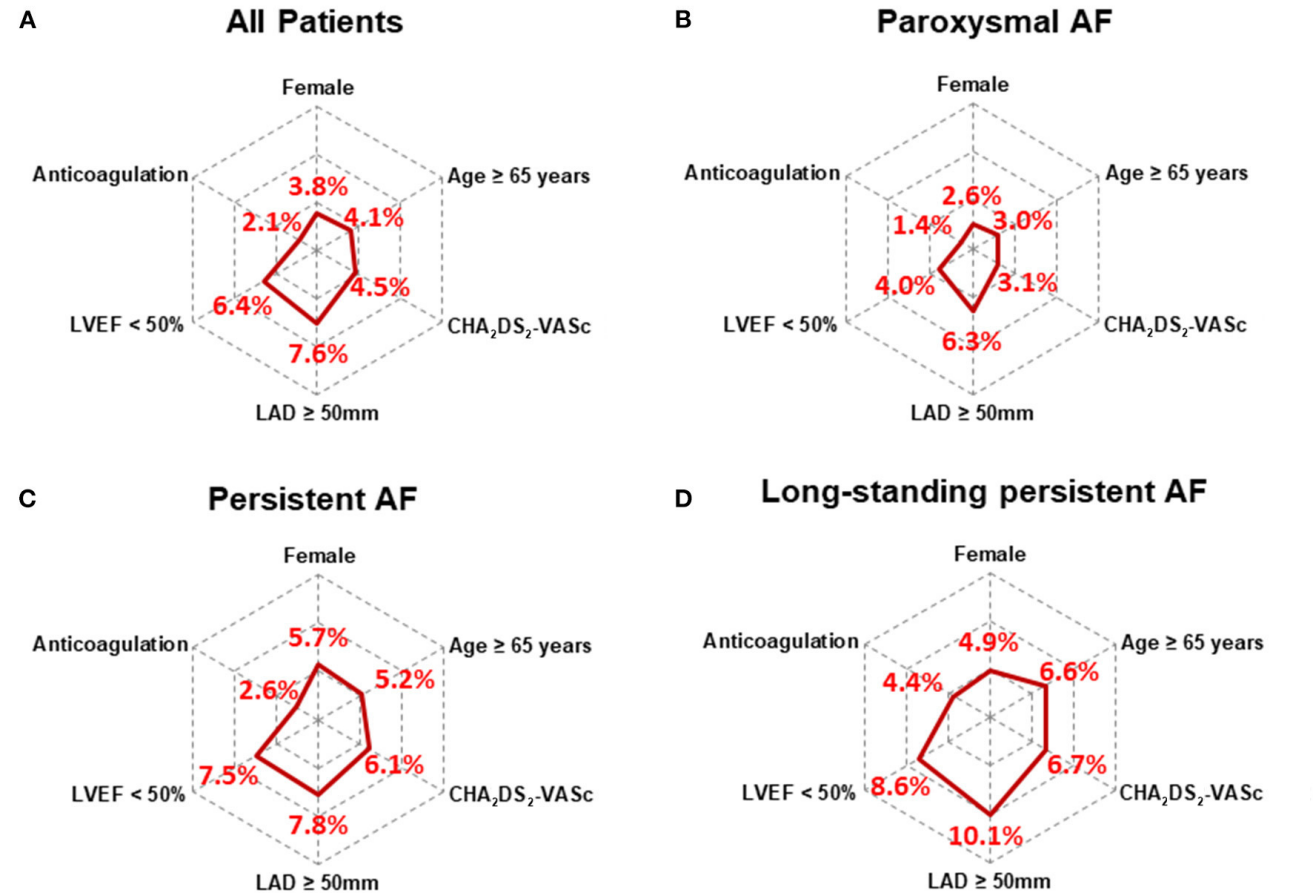
Radar chart showed the prevalence of left atrial thrombus in AF patients with various clinical characteristics. The overall prevalence of LAT varies with clinical characteristics **(A)** Results of subgroup analysis, including gender (female), age ≥ 65 years, CHA_2_DS_2_-VASc ≥ 2, LAD ≥ 50 mm, LVEF < 50%, and anticoagulation, showed the patients with paroxysmal AF always had the lowest LAT prevalence **(B)**, and patients with long-standing AF **(D)** presented the highest LAT prevalence except for female subgroup, in which its prevalence was lower than patients with persistent AF **(C)**. AF, atrial fibrillation; CHA_2_DS_2_-VASc, Congestive heart failure, Hypertension, Age ≥ 75 years, Diabetes mellitus, Stroke, Vascular disease, Age 65–74 years, Sex category (female); LAD, left atrial diameter; LVEF, left ventricular ejection fraction.

A total of 545 (2.7%) patients with paroxysmal AF had LAT, and the prevalence increased to 5.7% (808) and 6.8% (114) in patients with persistent AF and long-standing persistent AF, respectively (*P* < 0.001, Figure 2). LAT was present in 2.4%, 3.2%, and 4.5% of patients with CHA_2_DS_2_-VASc scores 0, 1, and ≥2 (*P* < 0.001, Figure 2). Patients administrated without anticoagulants had more prevalent LAT than those with anticoagulation (5.0 vs. 2.1%, *P* < 0.001) in overall, either in three AF pattern subgroups (paroxysmal:3.2 vs. 1.4%, *P* < 0.001; persistent:7.3 vs. 2.6%, *P* < 0.001; and long-standing persistent: 8.4 vs. 4.4%, *P* = 0.001). The overall prevalence of LAT vary with clinical characteristics (Figure 3A). The subgroups analysis, based on various clinical characteristics, including gender (female), age ≥ 65 years, CHA_2_DS_2_-VASc ≥ 2, LAD ≥ 50 mm, LVEF <50%, and anticoagulation, showed that the patients with paroxysmal AF always had the lowest LAT prevalence (Figure 3B), and patients with long-standing AF presented the highest prevalence of LAT except for female subgroup, in which the prevalence of LAT was lower than patients with persistent AF (Figures 3C,D).

### Risk Factors Associated With LAT

**Figure 4:**
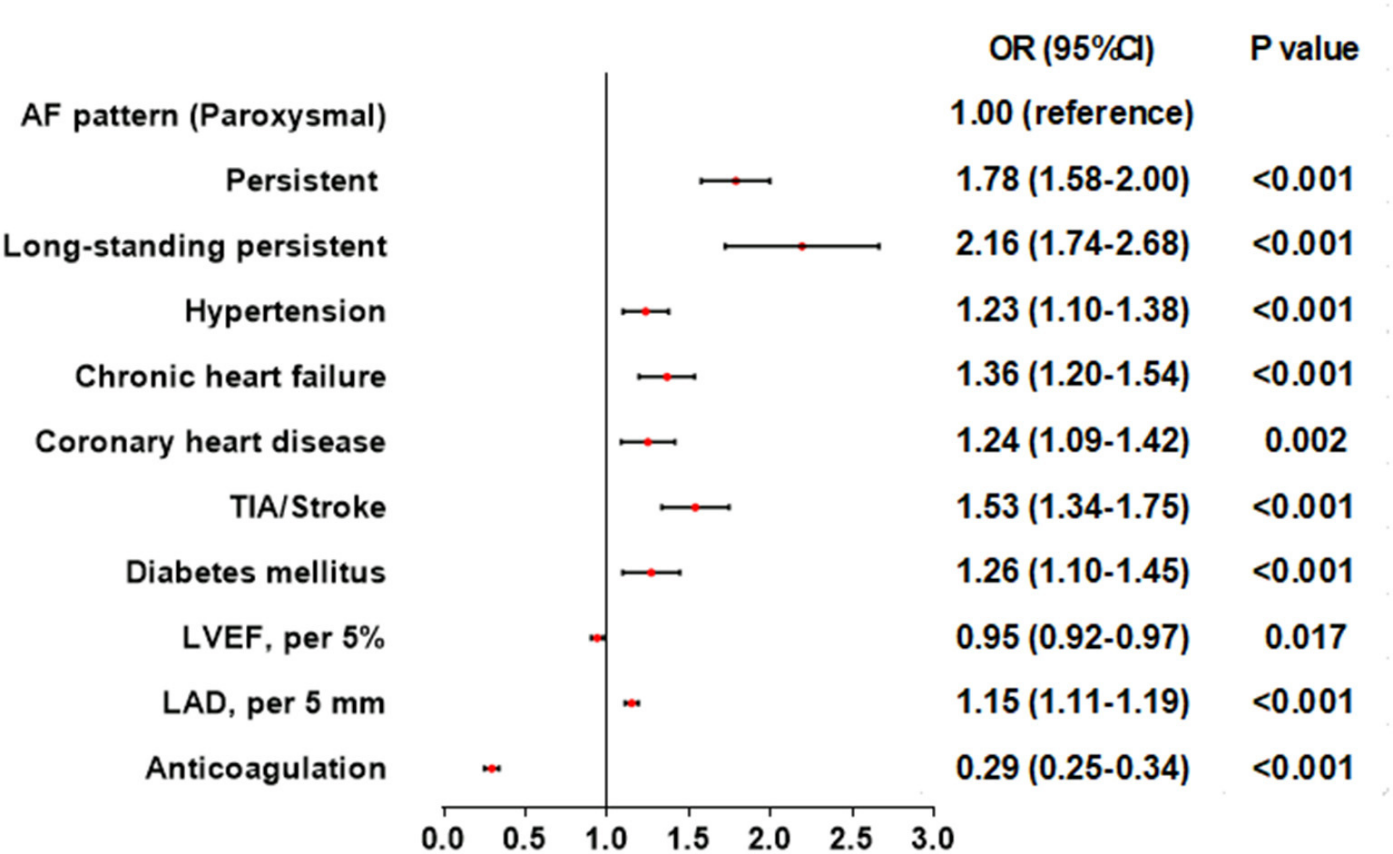
Association of patient characteristics and left atrial thrombus in multivariate logistic regression models. AF, atrial fibrillation; CI, confidence interval; LAD, left atrial diameter; LVEF, left ventricular ejection fraction; OR, odd ratio; TIA, transient ischemic attack.

After adjusting for confounding factors, including age, gender, hypothyroidism, hyperthyroidism, COPD, the stepwise regression model displayed that AF pattern [paroxysmal: reference; persistent: odds ratio (OR) 1.78, 95% confidence interval (CI) 1.58–2.00, *P* < 0.001; long-standing persistent: OR 2.16, 95% CI 1.74–2.68, *P* < 0.001], hypertension (OR 1.23; 95% CI 1.10–1.38, *P* < 0.001), chronic heart failure (OR 1.36, 95% CI 1.20–1.54, *P* < 0.001), coronary heart disease (OR 1.24, 95% CI 1.09–1.42, *P* = 0.002), TIA/stroke (OR 1.53, 95% CI 1.34–1.75, *P* < 0. 001), diabetes mellitus (OR 1.26, 95% CI 1.10–1.45, *P* < 0.001), and LAD (per 5 mm, OR 1.15, 95% CI 1.11–1.19, *P* < 0.001) were associated with an increased prevalence of LAT (Figure 4). However, it was demonstrated that higher LVEF (per 5%, OR 0.95, 95% CI 0.92–0.97, *P* = 0.017) and anticoagulation (OR 0.29, 95% CI 0.25–0.34, *P* < 0.001) were associated with a reduced prevalence of LAT (Figure 4).

## Discussion

To the best of our knowledge, the present analysis represents the largest study of the prevalence and characteristics of LAT in NVAF inpatients underwent TEE in China. The major findings include: first, the prevalence of LAT in inpatients with NVAF was 4.1%; second, LAT was more prevalent in patients with long-standing persistent AF, followed by patients with persistent and paroxysmal AF, and anticoagulation significantly reduced the total LAT prevalence by 58%; third, non-paroxysmal AF, cardiovascular diseases, diabetes mellitus, and larger LAD were associated with an increased prevalence of LAT, but the prevalence of LAT decreased with higher LVEF and anticoagulation.

Systemic and cerebral thromboembolism is one of the most feared complications for AF patients, and assessment of LAT is an important step in determining the management strategy for AF patients. It is well considered that LA/LAA is the main origin of embolism in patients with AF (12). The mechanisms underlying LAT have not been fully clarified, and multiple factors are involved in thrombosis, including blood stasis, endothelial dysfunction, and prothrombotic state. The different imaging modalities had varying sensitivity and specificity rates for LAT detection. Generally, cardiac magnetic resonance imaging and cardiac computed tomography have more advantages in detecting LAT than TEE (13, 14). However, both modalities are limited by local expertise and availability in comparison to TEE, and TEE is still the gold standard for the evaluation of LAA stasis and thrombosis (15). Thus, TEE remains the most widely used method to evaluate LAA thrombi prior to restoration of sinus rhythm in patients with AF.

In the present study, we retrospectively analyzed all NVAF patients undergoing TEE from a RWS-CAF. We found that the prevalence of LAT was 4.1%, which was lower than the pooled prevalence of 9.8% found in a previous study, but significantly higher than the prevalence among patients scheduled for AF ablation (16, 17). These differences from previous reports could be due to varying enrolled patients as well as different distributions of risk factors. The huge difference in LAT prevalence may also be attributed to the lower rate of anticoagulation treatment in China than in Europe and North America (18–21). Only 30.9% of included patients administrated with anticoagulants, although 72.0% of patients has a CHA_2_DS_2_-VASc Score ≥2. Furthermore, the heterogeneity on gene polymorphism, oral anticoagulation adherence, socioeconomic factors may ultimately affect the formation of LAT in various population (22). In conclusion, as we report the results of a real-world patient population, we believe that the present findings represent a typical patient population encountered in daily clinical practice in China.

Overall, patients with LAT presented more non-paroxysmal AF and cardiovascular comorbidities, and enlarged left atrium. Although the current management guidelines do not list AF pattern as a risk factor affecting the probability of LAT and stroke, several reports have revealed that non-paroxysmal AF carries a higher risk for thromboembolism and stroke than paroxysmal AF (5). In our study, the prevalence of LAT in paroxysmal AF was consistently lower than that of persistent and long-standing persistent AF, regardless of the stroke risk stratification assessed with the CHA_2_DS_2_-VAS_C_ score, age, gender, LAD, LVEF, and anticoagulation. Generally, advanced age has been considered an important risk factor for stroke in AF patients. However, old age has been found no association with an increased prevalence of LAT. The relative reasonable explanation may be that elderly people have a higher anticoagulation rate and treatment adherence than those in younger people (23, 24).

Consistent with previous reports (21, 25), the present study showed that cardiovascular diseases, higher LAD and lower LVEF were important predictors of LAT. However, a previous retrospective study in a cohort of 2,695 Chinese subjects showed that the CHA_2_DS_2_-VAS_C_ score was not significantly associated with LAT in patients with NVAF (26). These different results could be explained by varying proportions of high CHA_2_DS_2_-VAS_C_ scores. The proportion of CHA_2_DS_2_-VAS_C_ scores ≥ 2 was 41.3% in their study and 72.0% in our study, respectively. Ayirala et al. (27) demonstrated that both a higher LA volume index and lower LVEF are associated with LAA thrombus, with a ratio of LVEF to LA volume index ≤ 1.5 being highly sensitive. Doukky et al. (28) found that the parameters of LV diastolic function were also associated with LAA thrombus in patients with NVAF, and both a high E/e' ratio and low lateral e' velocity provided independent predictive value beyond the CHA_2_DS_2_-VAS_C_ score. LA enlargement will lead to a decrease in LA emptying and turbulent blood flow and then cause LA blood stasis, manifesting as LAT formation.

Compared with previous studies conducted in China, our research presented prominent feature and several novel findings. In simply, first of all, a large sample size and diverse data sources is the prominent feature in our study, which determined that our findings could be better represent the characteristics of AF inpatients in China. Second, real clinical data rather than subgroup screening data could show the full picture of inpatients with AF, and more comprehensively analyzed the relationship between factors and LAT under the perspective of clinical practice. In conclusion, we found the overall rate of LAT was not low in inpatients with NVAF in China.

## Limitations

Although the present results are conducted based on available, large sample, real clinical data from China, there were several limitations in our study. First, the observational nature does not allow drawing causal inferences. Second, the multicenter, retrospective study inevitably coexisted with heterogeneity in the patient population, detection methods and diagnostic standards, which may limit the generalizability of our findings. Third, the main limitation of our study is that its primary endpoint was the presence of LAT but not ischemic stroke. Although LAT formation is considered the main cause for thromboembolic events and appropriate risk stratification of AF patients, a study setting stroke as the clinical endpoint would be needed and designed in the further study. Fourth, the selection bias is inevitable, because the decision to perform TEE was based on clinical need, mainly for ruling out thrombus before surgery (such as catheter ablation, left atrial appendage occlusion, cardiovascular surgery, et al.) or electrical cardioversion. Which were not based on a serially recruiting clinical trials. Therefore, the study results need to interpret carefully and represent NVAF inpatients who underwent TEE would be more reasonably. Finally, many biological characteristics, socioeconomic factors, and pharmacokinetics are potentially associated with thrombosis in patients with atrial fibrillation, which are worthy of further study in the future, such as the relationship between LAT and types of anticoagulants, treatment adherence, genetic variation.

## Conclusions

In a real clinical population of inpatients with NVAF who underwent TEE examination in China, the prevalence of LAT was approximately 4.1%. In addition, non-paroxysmal AF, cardiovascular diseases, diabetes mellitus, enlarged LAD, lower LVEF, and lack of anticoagulation therapy were proven to be associated with LAT, and might improve thromboembolic risk stratification. Finally, more attention should be paid to the detection of LAT in NVAF patients with the above clinical characteristics.

## Data Availability Statement

The original contributions presented in the study are included in the article/supplementary materials, further inquiries can be directed to the corresponding authors.

## Ethics Statement

This study was approved by the Institutional Committee on Human Research at Renmin Hospital of Wuhan University (Wuhan, China). Written informed consent for participation was not required for this study in accordance with the national legislation and the institutional requirements.

## Author Contributions

SS, QZ, HH, and CH designed the research, performed research, analyzed and interpreted the data, performed statistical analysis, and drafted the manuscript. TL, SZ, and JL performed research, analyzed and interpreted data, and drafted the manuscript. SS, YT, and BY collected data. SS, QZ, and HH performed statistical analysis and drafted the manuscript. All authors have approved of the final draft of the manuscript.

## Funding

This research was supported by Grants from the Nature Science Foundation of China (Nos: 81800447 and 82170316), and the Nature Science Foundation of Hubei province (No: 2017CFB204).

## Conflict of Interest

The authors declare that the research was conducted in the absence of any commercial or financial relationships that could be construed as a potential conflict of interest.

## Publisher's Note

All claims expressed in this article are solely those of the authors and do not necessarily represent those of their affiliated organizations, or those of the publisher, the editors and the reviewers. Any product that may be evaluated in this article, or claim that may be made by its manufacturer, is not guaranteed or endorsed by the publisher.
